# Accelerated rTMS: A Potential Treatment to Alleviate Refractory Depression

**DOI:** 10.3389/fpsyg.2018.02017

**Published:** 2018-10-31

**Authors:** Chris Baeken

**Affiliations:** ^1^Department of Psychiatry and Medical Psychology, Ghent University, Ghent, Belgium; ^2^Ghent Experimental Psychiatry (GHEP) Lab, Ghent University, Ghent, Belgium; ^3^Department of Psychiatry, Vrije Universiteit Brussel – Universitair Ziekenhuis, Brussels, Belgium

**Keywords:** accelerated rTMS, HF-rTMS, theta-burst stimulation, major depressive disorder, refractory MDD

## Abstract

Three decades of clinical research on repetitive transcranial magnetic stimulation (rTMS) has yielded one clear treatment indication in psychiatry for major depression disorder (MDD). Although the clinical response equals the standard treatment algorithms, the effect sizes on the beneficial outcome remain rather modest. Over the last couple of years, to improve the efficacy in resistant depression, two new avenues have been developed: personalization and intensifying rTMS treatment. For the latter, we retrospectively compared accelerated high-frequency rTMS (arTMS) with accelerated intermittent theta burst stimulation (aiTBS) in the refractory depressed state. Although the clinical efficacy was not significantly different between both protocols, our observations substantiate the potential of the accelerated stimulation to shorten the treatment duration from the depressed state to the response state. Any time gain from the depressed state to the recovered state is in the patients’ interest.

## Introduction

Three decades of clinical repetitive transcranial magnetic stimulation (rTMS) research has resulted in only one clear indication for the treatment of (moderate) medication-resistant major depression in the field of psychiatry, specifically when stimulating the left dorsolateral prefrontal cortex (DLPFC) (Lefaucheur et al., 2014). Despite the many parameter adjustments tested over the past years, the rTMS outcome rates remain rather modest (Brunoni et al., 2017), meaning that only 3 or 4 out of 10 patients undergoing rTMS are going to respond, but will not be necessarily remitted. Importantly, like electroconvulsive therapy (ECT), another widely used neurostimulation application for treating patients with major depressive disorder (MDD), the rTMS must also be considered as an acute treatment option with relatively fast but no definitive long-term clinical improvement. Electroconvulsive therapy is considered to be one of our most potent treatment modalities in the acute phase of depression, yielding “unchallenged” response rates when compared with the classic pharmacological and current rTMS treatment algorithms (Baeken et al., 2013). After approximately 10 sessions of unilateral or bilateral (bifrontal/bitemporal) ECT, 80–90% of the treated depression patients will clinically respond (Kennedy and Giacobbe, 2007). Of note, when including the treatment-resistant depression (TRD) patients, the response rates tend to be a bit lower, which is around 50–70% (Rush and Siefert, 2009).

Over the last couple of years, to optimize the rTMS treatment efficacy, the individualization of rTMS parameters (Padberg et al., 2017) and intense rTMS paradigms are under investigation (Holtzheimer et al., 2010). For the first approach, to accurately target the stimulation area, individual anatomical data are included (Fitzgerald et al., 2009), individual brain connectivity patterns are selected (Fox et al., 2012), or depression “biotypes” based on the brain network analysis are targeted (Drysdale et al., 2017). The second approach, accelerated rTMS (arTMS) treatment, applies multiple daily sessions, reducing the total time period of stimulation by just over a couple of days. This is in contrast to the classical daily single rTMS sessions, which usually spread over 2–4 weeks and sometimes even longer (Holtzheimer et al., 2010; Baeken et al., 2015). It is of interest to note that when applied to the left DLPFC, no significant differences in remission or response rates or reductions in depression severity scores were observed between the accelerated and the classical daily rTMS treatments (Fitzgerald et al., 2018).

Newer stimulation parameters, which deliver pulses even on a shorter amount of time, compare the excitatory stimulation protocols of high-frequency rTMS (HF-rTMS) and intermittent theta-burst stimulation (iTBS), when applied to the dorsomedial prefrontal cortex (dmPFC). Bakker et al. (2015) did not find any differences in clinical outcome between these two protocols. Recently, in a large, randomized, non-inferiority trial with TRD patients, Blumberger et al. (2018) also reported no differences in the clinical outcome between HF-rTMS and iTBS treatments, when applied to the left DLPFC.

From a clinical perspective, it would be highly interesting if we could apply “intensified” rTMS treatment algorithms, resulting not only in fast clinical improvement but also in reaching the response and remission rates as observed with ECT. As mostly refractory depression patients are selected for ECT (besides psychotic and catatonic depression), we retrospectively compared our aHF-rTMS with accelerated iTBS (aiTBS) treatments in this research (Baeken et al., 2013; Duprat et al., 2016), by only selecting stage III TRD patients. We expected that in this refractory MDD sample, response and remission rates would not be significantly different between the patients receiving aHF-rTMS or aiTBS treatments. We also evaluated on the assumption that arTMS treatment would reach comparable remission rates [remission defined as a final score on the 17-item Hamilton Depression Rating Scale score ≤ 7 (HDRS: Hamilton, 1967)], as observed with ECT.

## Materials and Methods

From the original samples of our former aHF-rTMS and aiTBS accelerated paradigms comprising of patients with varying stages of treatment resistance (Baeken et al., 2013; Duprat et al., 2016), we have included only rTMS naïve unipolar stage III TRD patients in this research (Rush et al., 2003), with a mean duration of their current depression episode of 6.26 years (*sd* = 8.29), confirming only to the refractory MDD (Ward and Irazoqui, 2010). As ECT non-response is a poor predictor of clinical response to rTMS, stage V TRD patients were not included (George and Post, 2011).

A total of 28 stage III TRD patients remained, with 14 in each group. According to the criteria of Rush et al. (2003), all patients had a minimum of two unsuccessful psychopharmaceutical treatment trials with a selective serotonin reuptake inhibitor (SSRI) or serotonin-norepinephrine reuptake inhibitor (SNRI), and one failed clinical trial with a tricyclic antidepressant (TCA). After a washout period, all patients were noted as antidepressant-free, at least 2 weeks before entering the studies. Exclusion criteria were current or history of epilepsy, neurosurgical interventions, metal objects in the brain, having a pacemaker, having had ECT, alcohol dependence, and suicide attempts within 6 months before the start of the study. Bipolar and psychotic depressed patients were also excluded. Following standard practice, clinical response was defined as a 50% reduction of the baseline 17-item HDRS score (Hamilton, 1967) by certified psychiatrists not related to the studies.

In both accelerated sham-controlled crossover protocols, all refractory MDD patients were randomized to receive either active or sham stimulation (20 sessions in total, 5 sessions per day for 4 days) at 110% of the resting motor threshold. In the following week, the order was reversed. For aHF-rTMS (20 Hz) treatment, patients received 40 trains of 1.9 s duration, separated by an inter-train interval of 12 s (1560 pulses per session) (Baeken et al., 2013). For the accelerated iTBS treatment trial, patients received 40 trains (1620 pulses per session) in 54 triplet bursts with a train duration of 2 s and a cycling of 8 s period (Duprat et al., 2016). In both studies, we targeted exactly the same region of the left DLPFC under MRI guidance.

## Results

**FIGURE 1:**
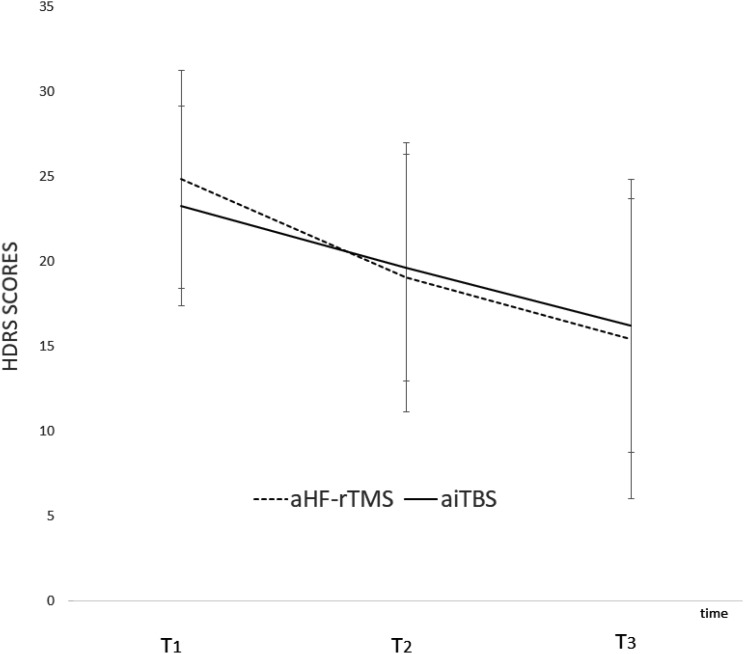
Graphical representation of the HDRS scores (mean and standard deviations) at baseline (T_1_), after 1-week (T_2_), and after 2-weeks of stimulation (T_3_) for aHF-rTMS and aiTBS treatment separately.

No significant differences on depression severity scores were observed between the two arTMS protocols. The 2 (Time; before vs. after treatment) × 2 (Group; aiTBS vs. HF-rTMS) × 2 (Order; first active then sham vs. first sham then active) analysis of variance (ANOVA) with individual HDRS scores as dependent variables showed a significant main effect of Time *F*(2,48) = 15.98, *p* < 0.01 but not of Group *F*(1,24) = 0.02, *p* = 0.88 or Order *F*(1,24) = 0.22, *p* = 0.64. The two-way interactions between Group and Order, Time and Order, and Time and Group were all not significant (*p*’s > 0.05). Also, the three-way interaction between Time, Order, and Group was not significant *F*(2,48) = 0.20, *p* = 0.82 (Figure 1). Separately mixed ANOVAs, separated for 1–2 weeks also showed non-significant results. Pearson *X^2^* showed no group differences in the remission rates [*X^2^*(1) = 1.12, *p* = 0.28]. Also, no significant demographic differences were observed. See Table 1.

**Table 1:** Demographic and outcome variables.

	All	aHF-rTMS	aiTBS	*p*-values
Number of patients	28	14	14	-
Gender (F/M)	14:14	8:6	6:8	0.45
Age	48.37 (11.74)	47.86 (13.58)	48.86 (10.05)	0.83
Response T_3_ (yes/no)	10	7	3	0.12
Remission T_3_ (yes/no)	4	3	1	0.28
Duration current depressive episode (years)	6.26 (8.29)	7.89 (8.03)	4.62 (8.51)	0.31
Order (R>S)	9:19	6:8	3:11	0.23
Benzodiazepine intake (number)	14	9	6	0.26
Benzodiazepine intake (mg/day)	10.11 (11.74)	11.71 (13.25)	8.50 (10.27)	0.48
rMT (%)	59.86 (6.27)	60.00 (4.98)	59.71 (7.54)	0.91
HDRS T_1_	24.07 (6.10)	24.86 (6.42)	23.29 (5.89)	0.51
HDRS T_2_	19.36 (7.21)	19.07 (7.95)	19.64 (6.69)	0.84
HDRS T_3_	15.82 (8.34)	15.43 (9.39)	16.21 (7.48)	0.81
HDRS T_4_	-	-	15.71 (8.23)	-
Response T_4_ (yes/no)	-	-	5	-
Remission T_4_ (yes/no)	-	-	3	-


*For these sham-controlled crossover studies, the 17-item HDRS was assessed at three time points. At baseline (T_1_), 3 days after the first round of (active or sham) accelerated 20 stimulation sessions (T_2_), and 3 days after the second round of 20 stimulation sessions (reversed order) (T_3_), the response is defined as at least a 50% reduction of the baseline HDRS score at T_3_. Remission is defined as having a final HDRS score of ≤ 7. T_4_ indicates the time of assessment for 2 weeks after the last measurement, after T_3_. These data were only collected for the aiTBS study.*

## Discussion

Aligned with the classical daily HF-rTMS/iTBS clinical outcome findings (Bakker et al., 2015; Blumberger et al., 2018), our observations with the accelerated aHF-rTMS/aiTBS treatment protocols suggest similar clinical effects. Although no significant differences between the two accelerated protocols were detected, 35% of the patients in the refractory depressed state responded, which was in line with the classical rTMS treatment response outcome for depression patients (Brunoni et al., 2017). For the remission rates, the 14% remission outcome seems rather low but is still in the range of results observed with the direct classical daily rTMS treatment, which is around 18.6% (Berlim et al., 2014). Of note, although the remission rates remained unchanged for 2 weeks after the acute treatment phase for aHF-rTMS (personal communication), the remission rates doubled after 2 weeks for aiTBS stimulation (Duprat et al., 2016), suggesting that the response and remission rates may be delayed for aiTBS. This is not contradictive to the neurophysiological effects, which are assumed to be induced by iTBS (Chung et al., 2015). In the current stage III study, as a result of adding the two remitters 2 weeks after having ended the aiTBS stimulation protocol (see Table 1), the remission outcome for both accelerated protocols was 21%.

It also seems that in the refractory depressed state, the response and remission rates are somewhat lower when including the less resistant depressed patients for the arTMS paradigms (Baeken et al., 2013: aHF-rTMS response: 35%, remission: 35%; Duprat et al., 2016: aiTBS response: 38%, remission: 30%), daily left DLPFC stimulation (Blumberger et al., 2018: HF-rTMS response: 47%, remission: 27%; iTBS response: 49%, remission: 32%), and daily dmPFC stimulation (Bakker et al., 2015: HF-rTMS response: 50.6% remission: 38.5 %; iTBS response: 48.5%, remission: 27.9%). However, given the limited number of large, placebo-controlled arTMS trials, this assumption is merely speculative at this point. Indeed, before taking firm conclusions for this retrospective stage III study, we clearly lacked sufficient power. To even find a small effect size of *f* = 0.10 in an ANOVA, for a *post hoc* calculation of repeated measures of ANOVA with a total sample of 28 patients, the power to find a group difference is 0.19, which is rather low (calculated with G-Power 3.1.9.2.). To properly address the question of whether arTMS paradigms could be as successful as ECT in refractory depression, large, multicenter, randomized, non-inferiority, placebo-controlled clinical trials will be necessary to compare arTMS with ECT in such samples. The absence of clinical differential effects between the sham and the active accelerated treatment suggests some form of placebo effect. Placebo responses in depressed patients undergoing rTMS have been reported earlier (Berman et al., 2000), and hopes and beliefs for a new treatment approach, after numerous failed pharmacological and psychotherapeutic interventions (Mommaerts and Devroey, 2012), may add to a placebo effect. On the other hand, a recent meta-analysis showed that the placebo effect may be a component of rTMS therapeutic response (Razza et al., 2018). Nevertheless, the standard contrasting to the accelerated left DLPFC and HF-rTMS treatment (Fitzgerald et al., 2018: standard response: 29.8%, remission: 17.5%; accelerated: 20.3%, remission: 11.9%) did not show any significant differences between the protocols, suggesting that standard daily and arTMS have similar clinical outcome rates in MDD.

Altogether, regardless of brain target, frequency, or coils, the response and remission rates do not seem to be that different between the rTMS paradigms. This also implies that within current safety parameters and stimulation guidelines, we may have reached a ceiling effect to treat depression, although with acceptable clinical results (compared to pharmacotherapy), but not with the outcomes of ECT. New types of coils, such as the H_1_ coil, as well as magnetic seizure therapy (MST) are used to improve the clinical outcome with some potential for higher remission rates (Harel et al., 2014; Cretaz et al., 2015), however, not with that magnitude to challenge the current ECT outcome rates. Individualization of the rTMS parameters has rightfully gained attention (Padberg et al., 2017) as Drysdale et al. (2017) were able to apply the machine learning algorithms (resting state fMRI), identifying the four biotypes of depression and increasing the prediction of response after dmPFC stimulation to 93.6%, when combining the connectivity and clinical features. Whether such scientific approaches will positively affect the future response and remission rates remains to be determined.

## Conclusion

Applying the current rTMS parameters for depression patients seems to yield similar response and remission rates, regardless of the stimulation protocols used. So the major impact of the arTMS protocols is the important gain of time. Instead of daily application for 4–6 weeks, similar results are to be expected when concentrating all sessions over a couple of days. From a patient as well as from a medical view, the restriction on the duration of the depressive episode may be a major motivation to consider arTMS. However, any rTMS treatment protocol has to be considered as an acute treatment, and the improvement and maintenance of clinical effects should remain as a priority in the field of neurostimulation, further pursuing clinical outcomes challenging the successes of ECT in refractory depression, especially for remission.

## Ethics Statement

The study were in accordance with the guidelines laid down in the Declaration of Helsinki (2004) and approved by the local ethics committee UZ Brussel and the local Ghent University Hospital. All subjects gave written informed consent.

## Author Contributions

CB collected and analyzed the data and wrote the manuscript.

## Conflict of Interest Statement

The author declares that the research was conducted in the absence of any commercial or financial relationships that could be construed as a potential conflict of interest.
